# Epidemiology of injuries in professional ice hockey: a prospective study over seven years 

**DOI:** 10.1186/s40634-020-00300-3

**Published:** 2020-11-06

**Authors:** Gregory Ornon, Jean-Luc Ziltener, Daniel Fritschy, Jacques Menetrey

**Affiliations:** 1 Centre de Médecine du Sport et de l’Exercice (CMSE), Swiss Olympic Medical Center, Hirslanden Clinique La Colline Geneva, Geneva, Switzerland; 2 Service de Chirurgie Orthopédique, University Hospital of Geneva, Geneva, Switzerland; 3 Service de Chirurgie Orthopédique, Hopital de La Tour, Meyrin, Switzerland

**Keywords:** Ice hockey, Epidemiology, Injury prevention, Concussion

## Abstract

**Background:**

Ice hockey injuries epidemiology is still poorly understood and very few studies are focused on it, especially about professional players.

**Methods:**

Our prospective study collected all injuries occurring on ice during practice and games over 7 years (2006–2013) in a professional hockey team playing in the 1st division championship in Switzerland.

**Results:**

During the 7 seasons, we recorded a total of 525 injuries and 190 injuries with time loss (TL).

Mean injuries incidence was 5.93 (95% CI 5.28 to 6.27) injuries/1000 h/player and with time loss 2.14 (95% CI 1.79 to 2.39) injuries/1000 h/player.

The lower limb was the most affected part of the body, with a total of 40.4% of all injuries, mostly knee Medial Collateral Ligament tear and muscle adductors/abdominal sprain. For the upper limb, shoulder was the most affected joint with mostly acromioclavicular sprain and shoulder dislocation.

Forwards had a significant (*p* < 0.05) higher risk than defensemen for knee Medial Collateral Ligament (MCL) tear. There was no significant difference in the concussion risk between forwards and defensemen, but defensemen had a significant higher risk (*p* < 0.05) to have a more severe concussion.

**Conclusion:**

This study provides a better understanding about professional ice hockey epidemiology, which is still insufficiently researched and understood. We also found some significant risk factors, being a forward for knee MCL tear, being a defensemen for concussion severity.

Concussion program prevention seems to be effective but it is crucial to continue the follow up of concussion on long term and expand the surveillance system to all the League.

## Introduction

Ice hockey is a team contact-sport with incredible levels of potential energy. Players have the possibility to skate at speeds up to 40 km/h [4], (among NHL data fastest speed is 40.9 km/h), allowing for aggressive and high-speed collisions with the other players. The pucks used can also travel at speeds well over 160 km/h [4, 7]. All of this, which takes place on the hard surface of ice, is paired with the static intervening obstacles on the field, such as the goals and the boards.

Despite well-developed protective gear, ice hockey can lead to high-energy collisions and traumas. Only few published studies have shown the injury epidemiology of ice hockey, and among those published, most focus on junior and/or elite teams [1, 4, 6–8, 10, 11] but not on professional teams.

This scientific study aims to collect data investigating the injury epidemiology, determining risk factors and effect of preventive measures within a professional ice hockey team over several seasons.

## Materials and methods

The study focus on a professional ice hockey team over a period of seven seasons (from 2006 to 2013). The team, over the period of study, took part in the National League (1st division in the Swiss Ice Hockey). The squad size remained relatively constant with around 25 players per season (with the exceptions of 24 players in 2007, 29 in 2011 and 31 in 2012). The ice hockey season is divided into three phases. Friendly games occurs in August and early September, regular season lasts from September to February, and the last part of the season depend the classification play off for top 8 teams and play out for the last 4 teams. This last part of season is a tournament, so it means the numbers of games can vary a lot from 4 to 21.

The injuries taken into account in this study all occurred on ice during training sessions or a games. Injuries must have required intervention of the team doctor during home games or intervention of the physiotherapist (who informed the doctor) during trainings or road games to be accounted for incidence. All data collected by doctors in a center based injury surveillance system on Excel spreadsheet with following details: type of injury, location of injury, date of injury, player position, cause of injury, medical diagnosis.

Injuries, which resulted in inability to play were referred as Time Loss (TL) injuries and were classified in 3 categories: minor (TL 1 to 7 days), moderate (TL 8 to 28 days), serious (TL > 28 days).

TL injuries which persisting after the end of the season were only included for the days of the season during which the athlete was incapable of playing.

Incidence rate was calculated using the following formula: Incidence = Number or injuries/(ice exposure x number of players) × 1000 expressed in units 1000 h/player. Exposure was defined and calculated as the following: Number of hours training on ice (usually 6 h per week) + number of hours played during games, we estimated 15 min per player/game without making difference between forwards and defensemen even if usually this playing time is slight greater for defensemen.

In this study will combine both injuries occurring during training and games to calculate overall injury incidence, without making the distinction between their respective injury incidence.

### Statistical analysis

All injuries were reported in Microsoft Excel spreadsheet. Descriptive analysis was performed also with Microsoft excel 2011. For statistical analysis, players were considered as Bernoulli variables with constant probability of injury/hour, plus creating a binomial distribution. Injury time loss followed a gamma distribution.

95% confidence interval (CI) is reported for injury rate. A *p*-value of 0.05 or less was set for statistical significance, and we used significance test.

## Results

During the 7 seasons, we recorded a total of 525 injuries and 190 injuries with time loss (TL).

Table 1 show injury incidence with and without time loss (TL) for each seasons.

**Table 1:** Summary of different Seasons and the respective injury incidence with and without time loss (TL)

Season	Total of injuries	Injuries with TL	Time loss (days)	Time loss/injury	Squad size	played hours	Incidence per 1000 h/player	Incidence with TL per 1000 h/player
**2006–07**	60	27	443	16.4	25	438	5.48 (95% CI 4.10 to 6.86)	2.47 (95% CI 1.54 to 3.39)
**2007–08**	82	34	415	12.2	24	521	6.56 (95% CI 5.12 to 7.97)	2.72 (95% CI 1.81 to 3.63)
**2008–09**	87	20	406	20.3	25	442	7.87 (95% CI 6.23 to 9.52)	1.81 (95% CI 1.02 to 2.60)
**2009–10**	85	25	578	23.1	25	556	6.12 (95% CI 4.82 to 7.41)	1.80 (95% CI 1.09 to 2.50)
**2010–11**	82	31	778	25.1	25	468	7.01 (95% CI 5.50 to 8.52)	2.65 (95% CI 1.72 to 3.58)
**2011–12**	68	32	978	30.6	29	482	4.86 (95% CI 3.71 to 6.02)	2.29 (95% CI 1.50 to 3.08)
**2012–13**	61	21	306	14.6	31	544	3.62 (95% CI 2.71 to 4.52)	1.25 (95% CI 0.71 to 1.78)
**Mean**	75.00	27.14	557.71	20.32	26.29	493.00	5.93 (95% CI 5.28 to 6.27)	2.14 (95% CI 1.79 to 2.39)

**Fig. 1:**
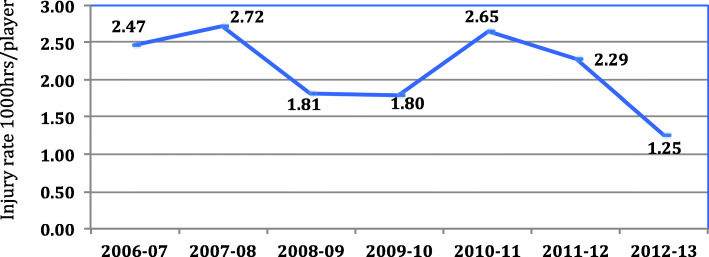
Evolution of Injury incidence with TL 1000 h/player during the study period

Mean injuries incidence was 5.93 (95% CI 5.28 to 6.27) per 1000 h/player and with time loss 2.14 (95% CI 1.79 to 2.39) per 1000 h/player. Figure 1 show the evolution of injury rate during the 7 seasons, the trend seems to show a decrease.

The incidence of injuries throughout the season can be seen in Table 2 (see below). August seems to be a high-risk period and responsible of 17% of all injuries with time loss and 22% of time loss, although no significant difference was found.

**Table 2:** Shows the amount of Time Loss (TL) Injuries per month during the 7 Seasons

	Forwards	Defensemen	Goalies	Total	%	Time loss (days)
August	25	8	0	33	**17.37%**	852
September	14	6	1	21	11.05%	378
October	21	10	1	32	**16.84%**	567
November	20	10	0	30	**15.79%**	835
December	10	9	1	20	10.53%	416
January	13	6	0	19	10.00%	440
February	15	6	0	21	11.05%	263
March	8	1	0	9	4.74%	124
April	5	0	0	5	2.63%	29
**Total**	**131**	**56**	**3**	**190**	**100.00%**	**3904**

**Fig. 2:**
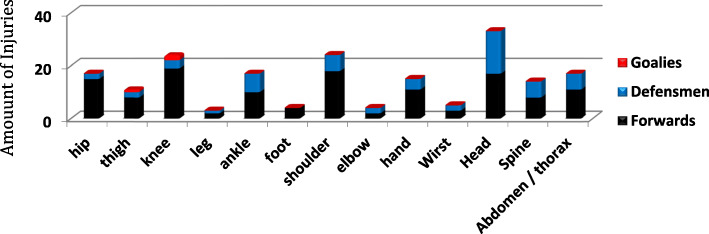
Injuries location with time loss 2006–2013

Results about injuries location show that the lower limb is most vulnerable to injury, representing 40.4% of total injuries. This compares to 25.5% for the upper limbs, 25% for the head and spine, and the remaining 9% for the abdomen and thorax. The two joints most significantly affected showed to be the knee (with MCL tears) and the shoulder (with acromioclavicular sprain and shoulder dislocation), both representing 12.8% of injuries. Figure 2 represents the distribution of injuries with TL around the body. Head, shoulder and knee injuries are leading to the majority of time loss.

### Type of injuries

Ligament affiliated injuries (sprain), including dislocations, represent the majority of all injuries with 33%. Ligament injuries also prove to be the most TL-injuries representing 40% of all time loss. Forwards had a significant (*p* < 0.05, significance test) higher risk than defensemen for knee Medial Collateral Ligament (MCL) tear.

**Fig. 3:**
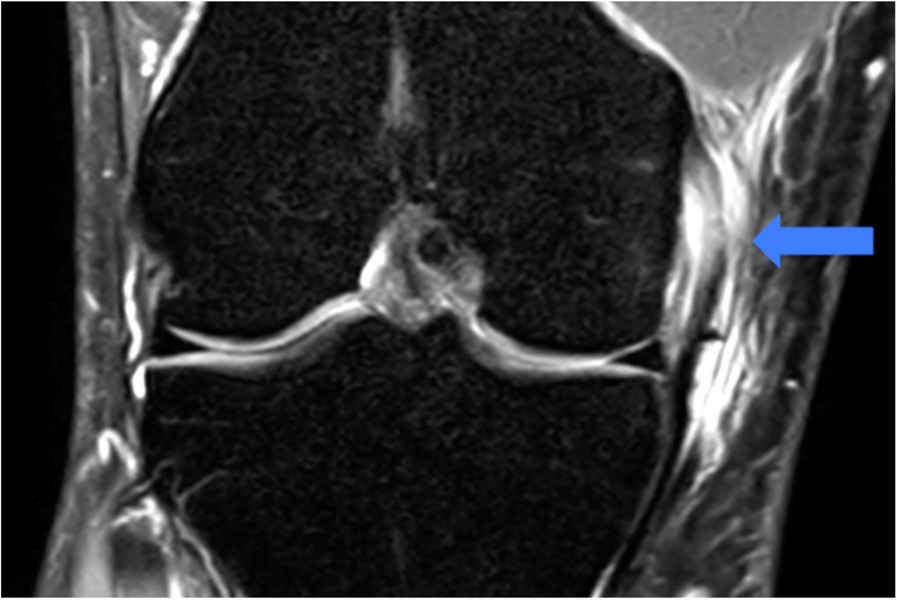
Knee Medial Collateral Ligament tear proximal, MRI T2 image with a proximal hypersignal of the MCL representing by the blue arrow

Figure 3 shows a typical injury in ice hockey: knee MCL tear.

You can see a MRI T2 image with a proximal hypersignal of the MCL (blue arrow).

**Fig. 4:**
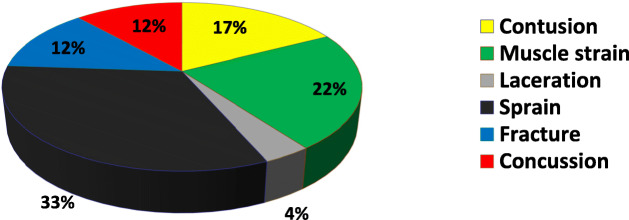
Injuries type distribution leading to TL

Figure 4 shows injuries distribution leading to time loss.

It should be noted that fractures although only consisting of 12% of injuries with TL but leading to severe injuries with 20% of all time loss. In comparison, contusions are more frequent than fractures but usually benign, leading to minor injuries and represents only 8.7% all time loss.

### Cause of injury

**Fig. 5:**
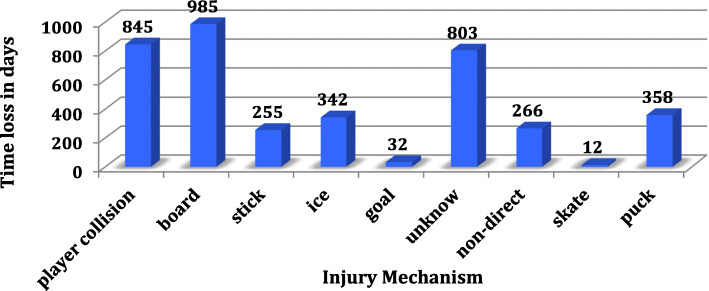
Time loss in days depending of the cause

Figure 5 shows the time loss depending the mechanism of the injury: player collision, board contact, hit by stick, fall on ice, collision with the goal, unknown, non-direct (no contact usually for muscle strain), cut by the skate, hit by the puck.

Identify one cause of injury was challenging as often multiple are responsible for each injury (For example, players contact followed by a collision into the boards). Therefore, only one cause was noted for each injury, choosing the most dominant cause leading to injury.

Interestingly, the cause of severe injuries (> 28 days off) is most frequently a collision with another player (28.6%), followed by a collision with the boards (19%) and then hits by the hockey stick (14.3%).

It should be noted that no data was collected for the first season (2006–07).

**Fig. 6:**
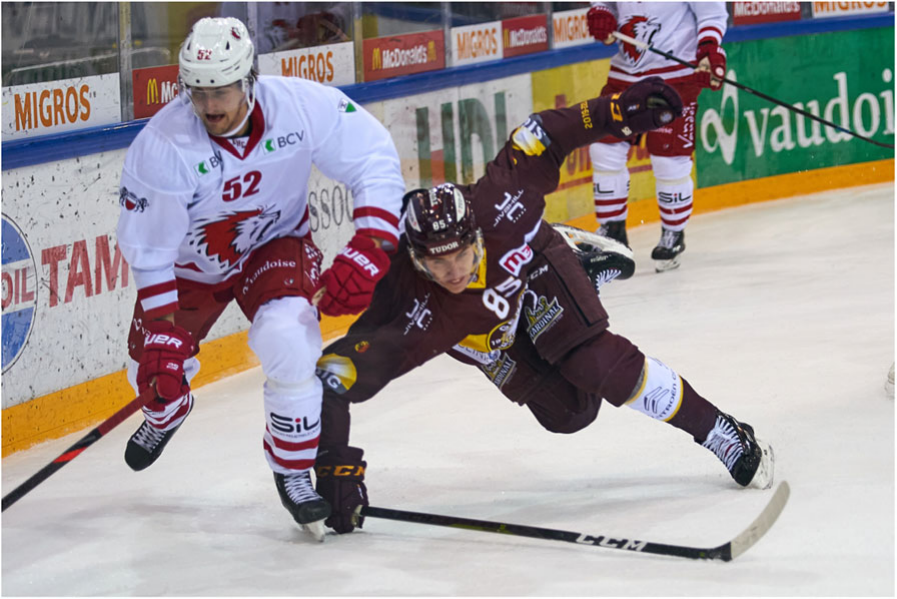
typical game situation with a collision between two players followed by a crash on ice

Figure 6 shows a typical game situation with a collision between two players followed by a crash on ice.

### Severity of the injuries

**Fig. 7:**
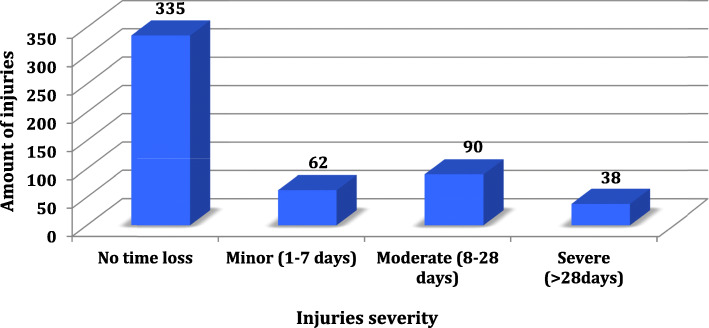
Injuries severity distribution during the 7 seasons

**Fig. 8:**
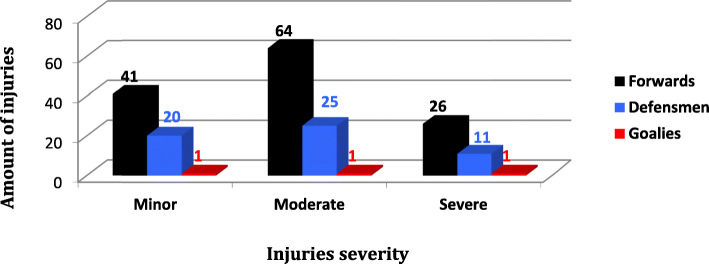
Injuries (with time loss only) severity distribution by player position

Figure 7 shows the distribution of all injuries (with and without TL) during the 7 seasons and Fig. 8 the injuries distribution only with TL by player position. Injuries without time loss are largely predominant as you can see in the graph 7.

In terms of minor injuries, the most frequent showed to be muscle-related (31%) and predominantly affected the forwards.

For moderate injuries, ligament strain were predominant (32%) with the most common diagnosis of knee MCL tear, affecting mostly the forwards (89%).

Concerning severe injuries, ligament-related injuries are also the most frequent with the most common diagnosis of shoulder dislocation.

The longest time loss (202 days) was due to a fracture of the patella which occurred at the beginning of the season and was treated surgically (but he resumed playing next season).

**Fig. 9:**
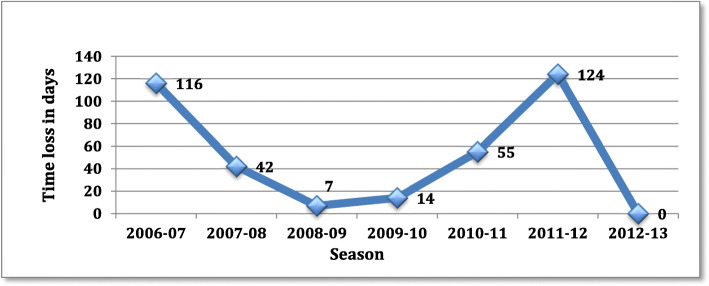
Time loss in days depending adductors and abdominal sprain

**Fig. 10:**
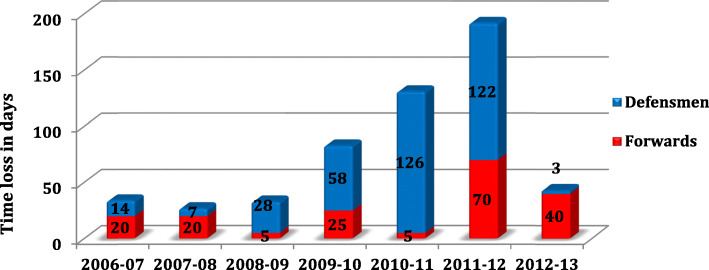
Time loss in days depending to concussion by position

Figures 9 and 10 shows the time loss depending respectively adductors/abdominal sprain and concussion.

Concussions, in our data, represent 15% of all injuries and are responsible of 14% of TL during the 7 seasons, it represents 543 days off and also the highest TL due to a diagnosis, MCL tear is in 2nd position with 456 days off.

There was no significant difference in the concussion risk between forwards and defensemen, but defensemen had a significant higher risk (*p* < 0.05, significance test) to have a more severe concussion.

## Discussion

In our study we reported an injury rate for the 7 seasons of 5.93 (95% CI 5.28 to 6.27) per 1000 h/player and with time loss 2.14 (95% CI 1.79 to 2.39) per 1000 h/player.

We demonstrated that forwards had a significant (*p* < 0.05) higher risk than defensemen for knee Medial Collateral Ligament (MCL) tear and defensemen had a significant higher risk (p < 0.05) to have a more severe concussion.

For both adductors/abdominal sprain and concussion, the introduction of preventive program (2007 for adductors and 2011 for concussions) seems to be effective, but longer follow up is needed.

Literature shows a large difference in injuries incidence, and vary from 2.3 to 79.2 injuries/1000player-hours [7]. Despite this discrepancy, all studies has shown that injury-risk is significantly higher (from 2 to 8 times) during games compared to training sessions, so we didn’t interest to this risk factor in our study.

Our injuries incidence is difficult to compare to others studies, as they combine large and diverse scopes of study. For example, three of the studies focus to junior American teams in the NCAA and three others to Swedish and Danish amateur teams. The time period for the studies range from 1 to 15 years and the number of teams followed also differ, from only one to many (10+). In addition, injury definition can differ (necessity of medical intervention, inability to play, season end).

Finally, an other factor is very hard to estimate precisely, the exposition of each player during a game.

Effective time on ice varies a lot from one player to the other (from few seconds up to 30 min) and can be only estimated. In our study we choose a mean of 15 min per player.

One study has a similar population about professional ice hockey players and collected data over 6 NHL seasons [7]. In this study they reported an injury rate of 49.4 /1000 h/player which is significantly higher than our rate. This is mostly due to the definition used for exposure time in this study. Unlike the NHL study, in our study time spent training was also included in the calculation, which represents 90% of the total exposure time on the ice. Therefore, as injury risk is substantially lower during training, the total injury risk, also including games, is lessened. If only games were taken into account, the injury incidence would be probably about 60.0 injuries/1000 h/player, closer to the NHL value of 49.4.

Comparing injury rate between different sports proves to be even more difficult than comparing different studies within the same sport. There exists a large range of possible data collection, methods for exposure estimation contributing to this challenge.

A study by Ekstrand [3], over 11 football seasons of the European championship league reports an injury incidence with time loss of 7.4/1000 h/player (both training and match play times included). This is largely greater than this investigation’s value of 2.29 injuries/1000 h/player.

Another study focused on a 1st division French rugby team [2], which collected data on all match-related injuries causing a minimum of 8 days off match play. The injury rate was 40.7 injuries/1000 h/position. This value, which seems significantly elevated, only takes into account injuries during match play. It has been consistently shown by other studies that match play causes greater injury risks than training does, supporting this result.

In handball, a prospective study in a club with junior and elite athletes [9] published a total injury incidence of 6.1 injuries/1000 h/player. The injury incidence was higher for elite players during matches at 22.2 injuries/1000 h/player. This accounted for any injury causing absence from training or game.

This investigation aimed to gain a more comprehensive understanding of injury rates and trends in injury severity in the sport of professional ice hockey. Injury incidence rates, interestingly, in ice hockey are not significantly higher than other team contact-sports (if training and match play included). For ice hockey, incidence rates show to be 2.29/1000 h/player, which compares to 7.6/1000 h/player for football [3] and 6.1/1000 h/player for handball [9].

Our results shows two injuries rate peaks during the season. The first in August, representing 17% of all injuries, and the second in October and November representing 16 and 15% of total injuries respectively.

The month of August represents in Switzerland the return on ice with training sessions and friendly games, usually called summer camp. This month seems to be a high-risk period with a lot of injuries and responsible of 852 days of time loss. This finding could be explained by the fact that no risk is taken to return to play in this period of friendly games. The second peak of injuries, around October/November, could be explain by the fact that games schedule is more intense during this period with up to 3 games/week, associated with less recovery time and logically increasing the injury risk. Injury rates decrease in December is probably due to a period of rest (10 days) over the holidays, also common during the months of March and/or April depending if the season has ended early due to poor results in play off.

Finally, we can note that the mean time loss per season for the entire squad is 524 days this representing 2.5 player positions in the squad or 10% of absenteeism.

Adductors and/or abdominal muscle tears were responsible of about a quarter of time loss injuries during the season 2006–2007. Since then, preventive measures were taken to reduce the occurrence of this type of injury. Such measures included: isokinetic tests to determine possible muscular imbalances, as well as introduction of strength and conditioning programs focusing on eccentric movements and specific stretching, during the off ice season. After integration of these programs, in 2007, time loss injuries drastically decreased across the successive seasons.

The new increase of adductors and/or abdominal muscle tears in 2011 could be explain by a lot of change in the squad, players, staff therefore, especially the physical conditioning trainer, potentially reducing the rigor with which the injury prevention programs were implemented. This might have consequently caused the injury spike during that season and further efforts were made to re-implement preventive measures against muscle injuries around the hip.

Ice hockey is also identified as a sport with high risk of concussion (also described in medical literature as minor head trauma). This is confirmed by our data, since concussion are responsible for 14% of time loss (513 days) and 12% of all injuries with time loss. This result is close from literature data where concussions represent 2–14% of all hockey injuries [5].

In our studies the time loss related to concussion is increasing constantly during the 6 first seasons.

Reasons for this increase could include a better medical understanding and recognition of concussions and an increased awareness of players, coaches and families. The increase in physical load during the games over the last decades could also play a role. Over the time, recovery and rehabilitation protocols were established with daily assessments of athletes to facilitate the return to play.

This increase could be similar of what happened in NHL after the implementation of the Concussion Program in 1997 [5]. This specific program was introduced to better integrate the SCAT in the NHL. Simultaneously, the prevalence of concussions increased, and also the time loss due to concussions.

A similar program was introduced in 2011 by the Swiss hockey League, called “Respect My Head”. The goal was to increase awareness of the risks surrounding concussions. This followed with stricter rules during games, especially concerning shocks to the head.

In our data, we can’t show a significant decrease of concussion rate and severity after the implementation of the campaign “Respect my head”, because a longer follow up is needed. However the trend in 2012 seems to show a decrease in concussion severity (6 concussions in 2011 leading to 192 days of time loss, 6 minor concussions in 2012 leading to 43 days of time loss). In the future, the goal will be to extend this injury surveillance system to the entire Swiss hockey league to appreciate the effect of prevention program. Reinforcement of the rules and respect among players are also two important factors to limit the risk of concussion.

Generally, a good central injury surveillance system seems essential to establish a greater understanding of hockey-specific traumas and to determine some risk factors and possible protective measures to prevent serious injury.

## Conclusion

This study provides a better understanding about professional ice hockey epidemiology, which is still insufficiently researched and understood. We also found some significant risk factors, being a forward for knee MCL tear, being a defensemen for concussion severity. This study has shown, that ice hockey is not responsible for significantly greater injury rates compared to other team contact-sports as football, rugby or handball.

Prevention program seems to be effective but it is crucial to continue the follow up on long term, especially for concussion and expand the surveillance system to all Swiss Hockey League in order to better understand injury risk factors leading to concussion. A long-term objective for a safer practice of professional ice hockey, using the results of this study, would be to standardize injury data collection methods for all Swiss professional hockey teams.

## Abbreviations

TL: Time Loss
MCL: Medial collateral ligament
